# Data on the assessment of Groundwater Quality in Gomti-Ganga alluvial plain of Northern India

**DOI:** 10.1016/j.dib.2020.105660

**Published:** 2020-05-07

**Authors:** Apoorv Verma, Brijesh Kumar Yadav, N.B. Singh

**Affiliations:** a Civil Engineering Department, Institute of Engineering and Technology, Lucknow-226021; b Department of Hydrology, Indian Institute of Technology, Roorkee-247667; c Harcourt Butler Technical University, Kanpur-208002

**Keywords:** Groundwater, Water Quality Index, Inverse Distance-Weighted, Irrigation indices

## Abstract

This data article deals with the assessment of groundwater quality based on water quality index (WQI) and irrigation indices. A total of 8 sites have been selected for the qualification of groundwater fitness. The assessment of groundwater quality has been done by selecting 13 physico-chemical parameters such as pH, EC, TDS, Ca^2+^, Mg^2+^, Na^+^, K^+^, Cl^-^, SO^4-^, HCO^3-^, NO^3-^, F^-^, and TH. Inverse distance-weighted (IDW) application was used to prepare the spatial distribution maps of WQI for the pre and post-monsoon period. All the samples were found in the rock dominance zone in Gibbs plot and according to the Piper plot, Ca-HCO_3_ is the dominant hydrochemical facies in the study area. On the other hand, irrigation water quality was examined by computing irrigation indices such as SAR, RSC, SSP, MHR, KR, %Na, PI, and PS. The outcomes of the irrigation indices suggests that the water quality is of a good and excellent category except for MHR and RSC.

Specification table

**Table:** 

Subject	Environmental science
Specific subject area	Water quality, groundwater management
Type of data	Table Figure
How data was acquired	Digital meter PC/301, CB18/945 Generic hand-held TDS-3 digital meter, Ion chromatography: Metrohmn 792B-IC, Arc GIS version 10.4.1, Origin 8.5-Data analysis and graphic software.
Data Format	Raw Analyzed
Parameters for data collection	A total of 13 physico-chemical parameters are selected (pH, EC, TDS, Ca^2+^, Mg^2+^, Na^+^, K^+^, Cl^-^, SO^4-^, HCO^3-^, NO^3-^, F^-^, and TH) to collect the dataset for analysis of groundwater quality.
Description of data collection	Samples were collected according to the standard procedure in 1L clean polyethylene bottles in June, 2015 (8 samples in pre-monsoon season) and January, 2016 (8 samples in post-monsoon season). Above mentioned chemical parameters in the abstract section were analyzed as per the standard method.
Data source location	Gomti-Ganga alluvial plain, Lucknow, Uttar Pradesh, India. The GPS coordinates of the sampling points are presented in Table 1.
Data accessibility	Data are included in this article

**Value of data**

• The data in this article gives an overview of groundwater quality that will help regulatory bodies and local authorities to improve and develop preventive measures for safe drinking and irrigation water use.
• This data article proven the implication of water quality indices that would be valuable for decision-makers and governing bodies to implement the appropriate management plan.
• The Gibbs plot can be used to understand natural groundwater chemistry and its control mechanisms. In addition, piper diagram help in determining the hydrochemical facies of groundwater.
• This data article can help to understand the ion exchange processes, the origin of ion elements and concentrations in groundwater in the study area.
• This regional-scale study in the field of groundwater quality can help in the management and mapping of groundwater on a high-resolution scale in a global way.

## 1 Data

**Fig. 1:**
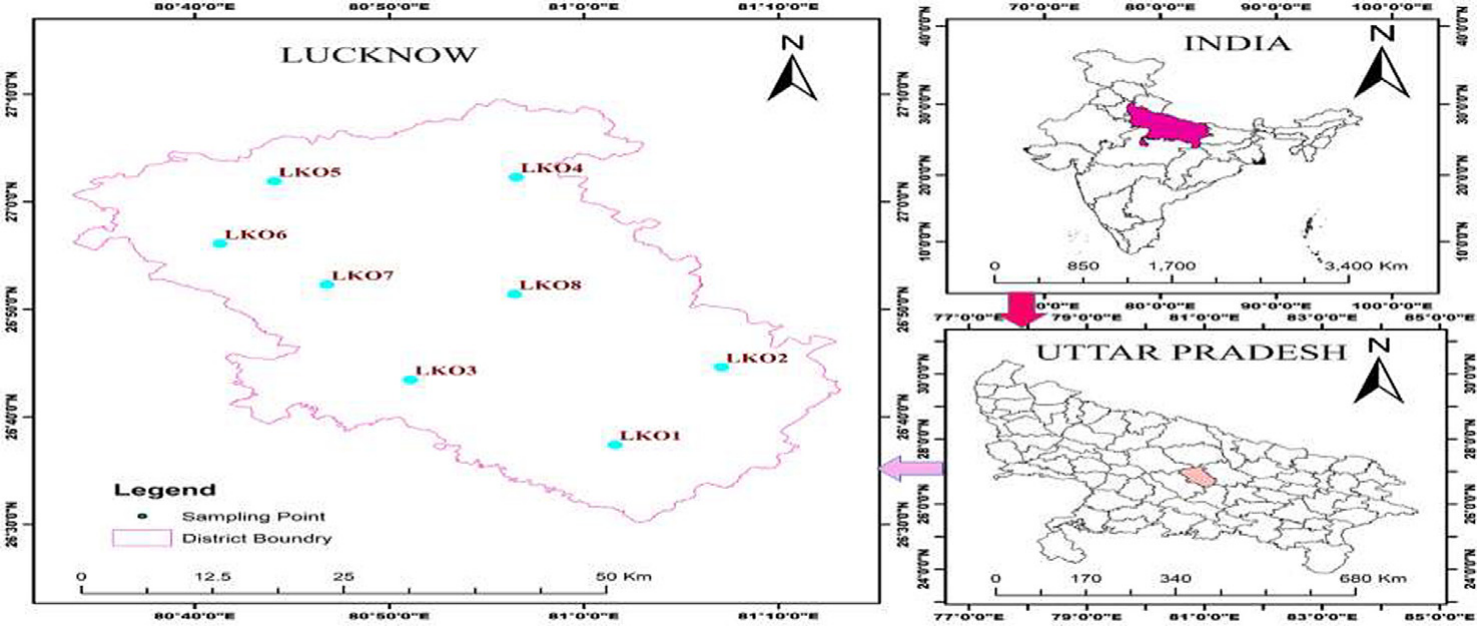
Location of study area and sampling points, Lucknow, India.

**Fig. 2:**
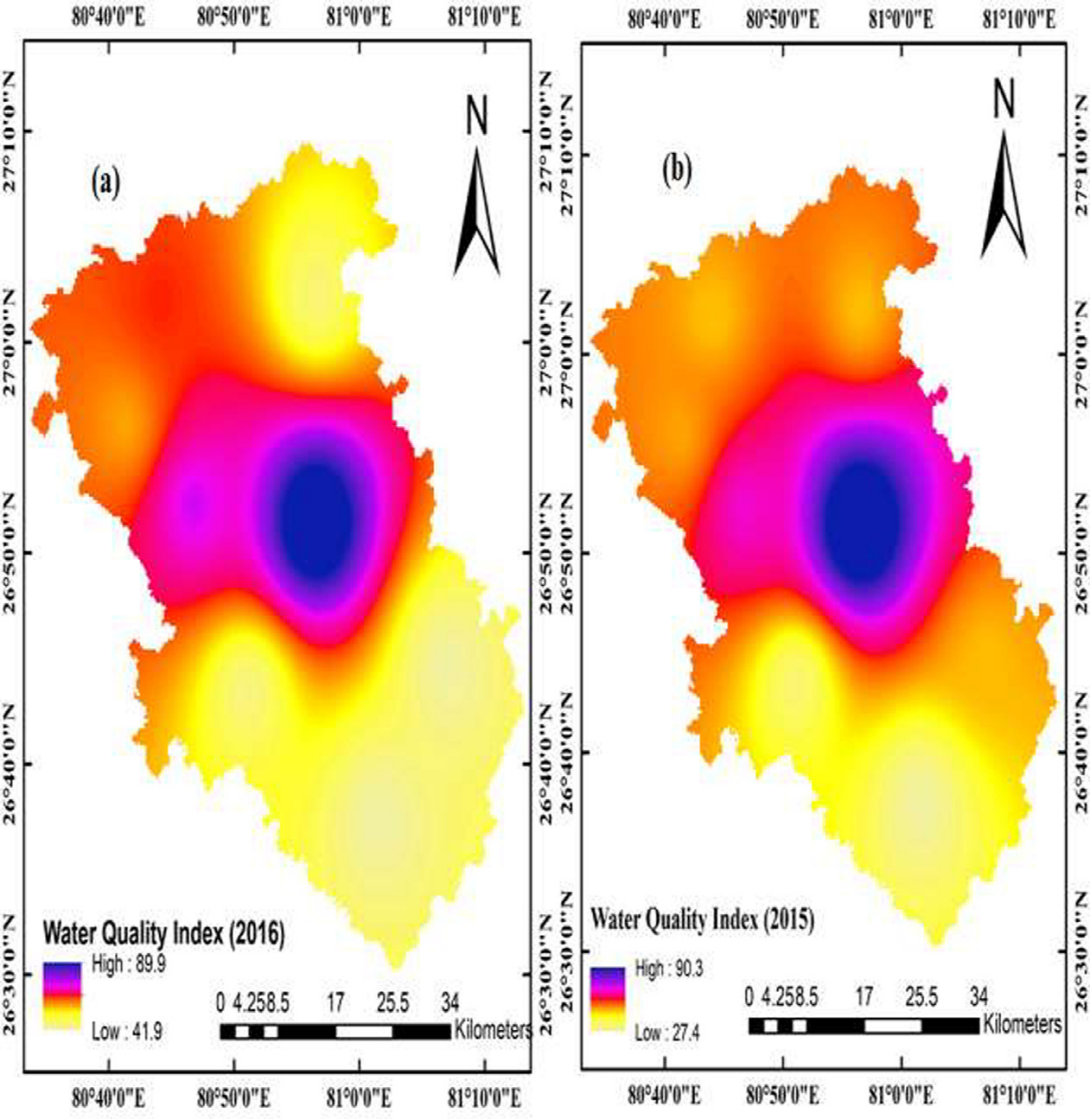
Spatial distribution map of groundwater quality index (a) 2016: Post monsoon (b) 2015: Pre monsoon.

**Fig. 3:**
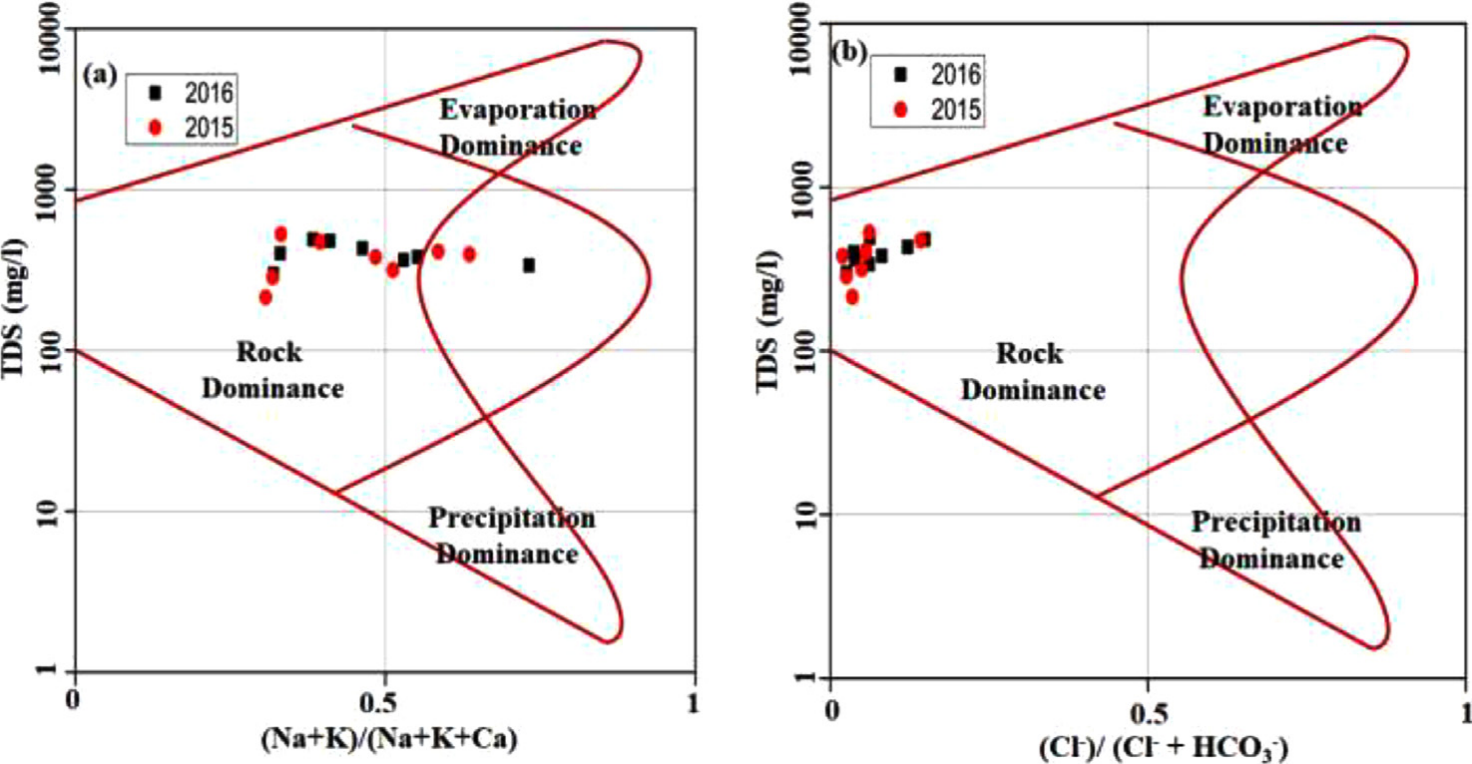
Gibbs plots a. TDS vs (Na + K)/ (Na + K+ Ca) b. TDS vs Cl (Cl + HCO_3_).

**Fig. 4:**
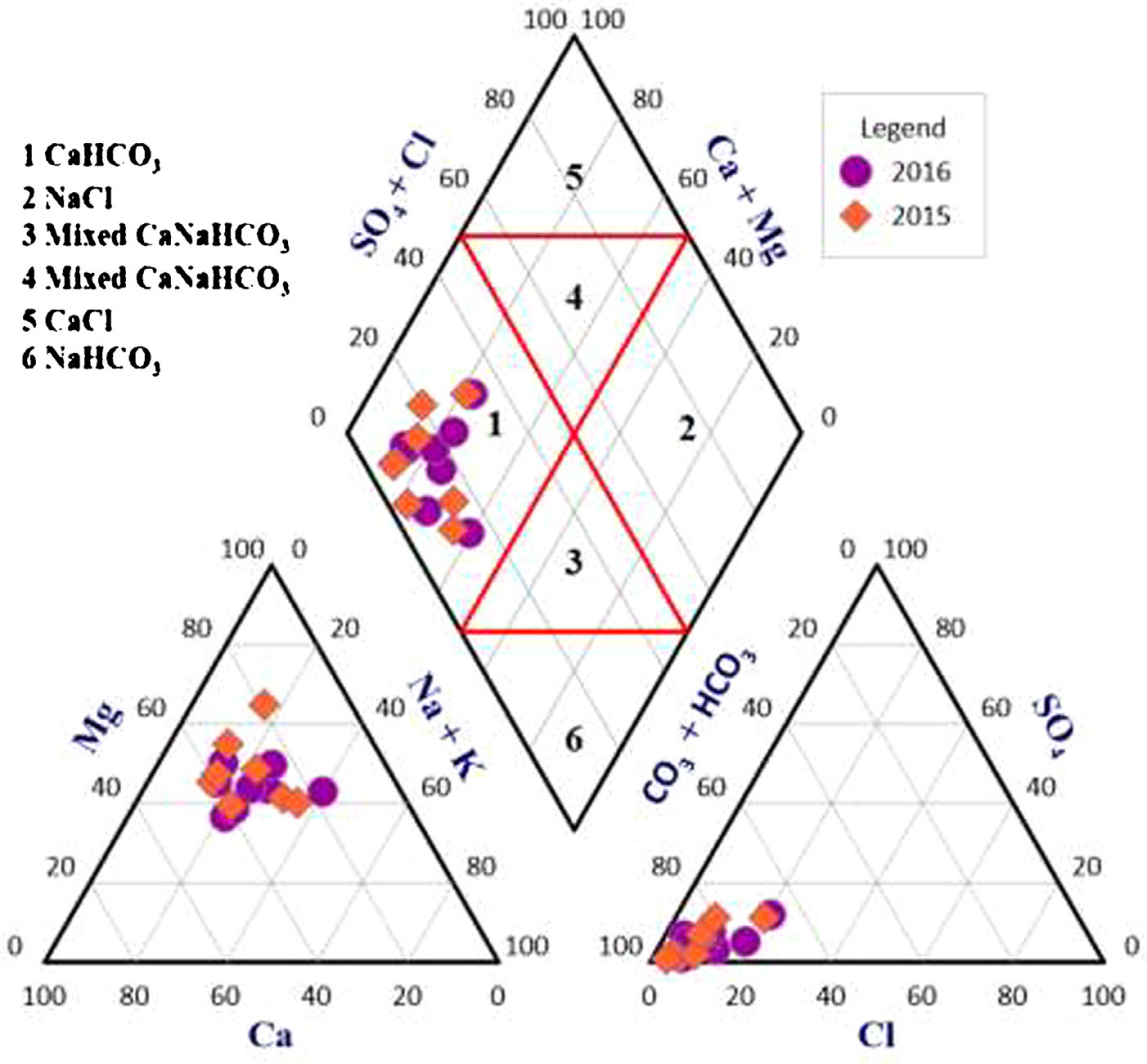
Piper's Trilinear plot of major ion data of groundwater samples.

**Fig. 5:**
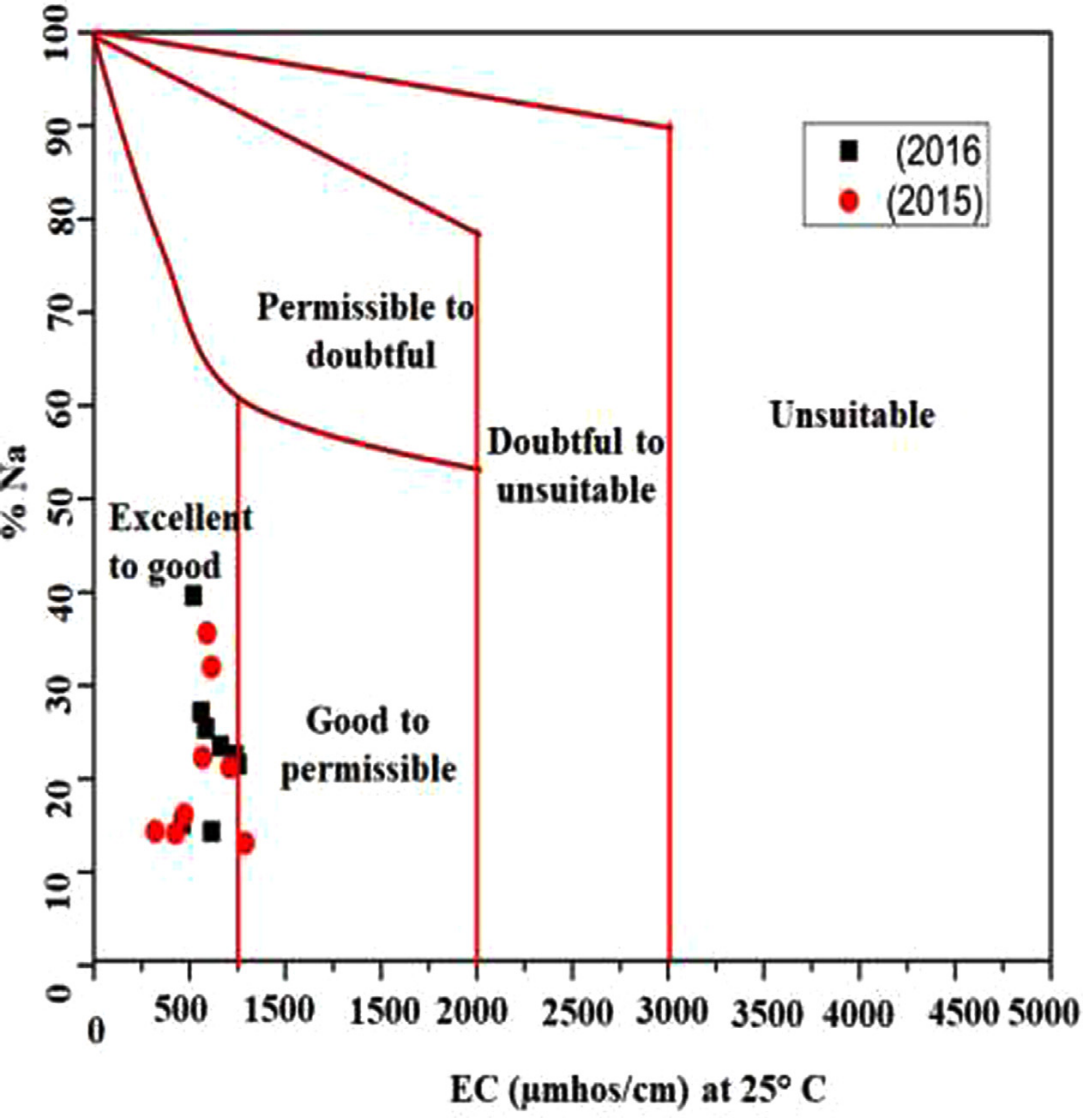
Wilcox diagram, EC vs % Na.

**Fig. 6:**
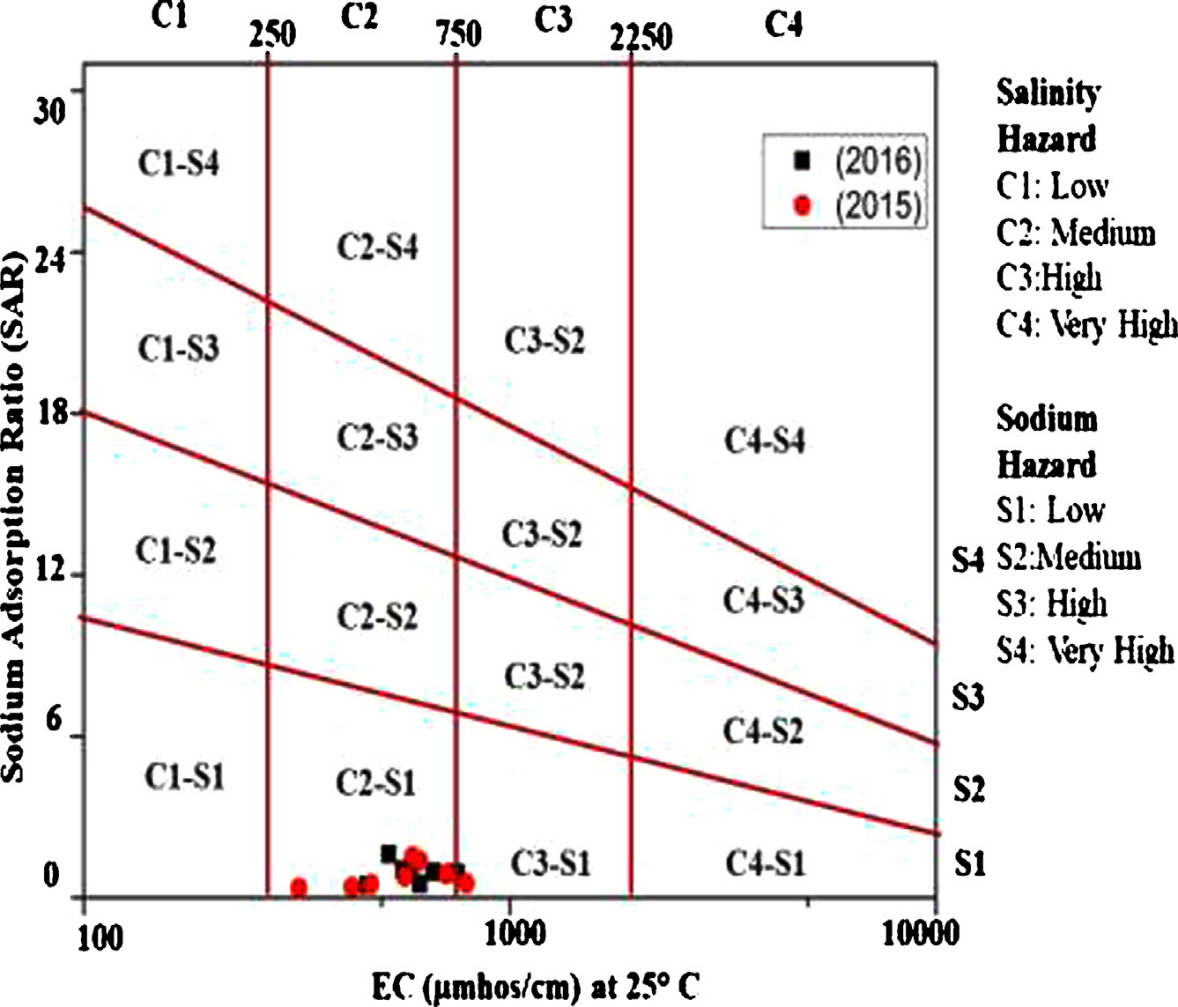
USSL diagram, Salinity Hazard (EC) vs Sodium Hazard (SAR).

**Fig. 7:**
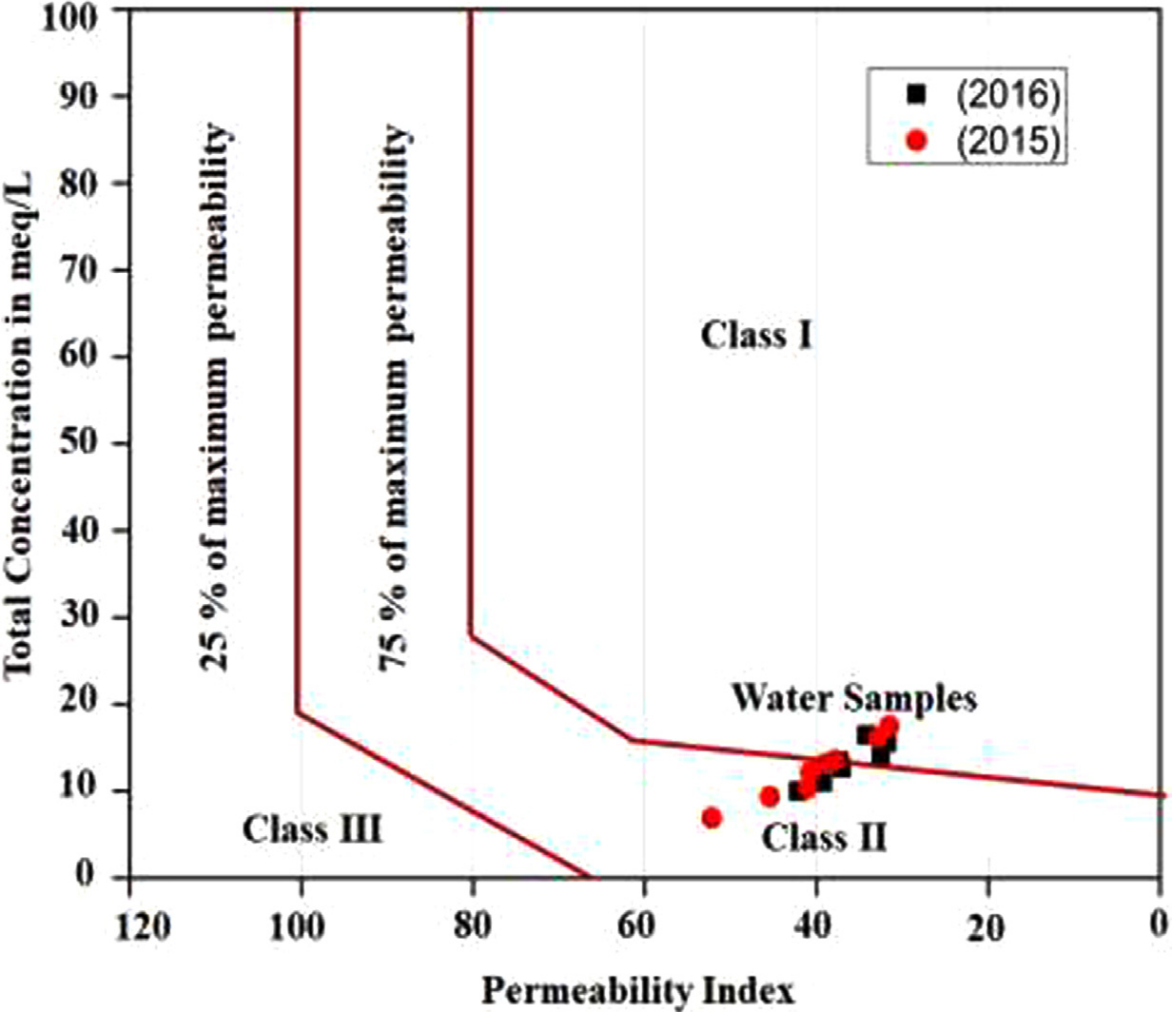
PI vs Total concentration (in meqL^-1^).

This data article contains 9 tables and 7 figures to describe the quality of groundwater used for drinking and irrigation. The accuracy of data is verified by calculating the percent charge balance error (%CBE) shown in Table 1. Fig. 1 represents the study area location along with sampling points. The field observations and laboratory analysis of physico-chemical data is presented in Table 2. The data from Table 2 are used to calculate the water quality index (WQI) which is summarized in Tables 3–5. Correlation analysis is a prevalent and widely used approach between hydro-geologists and environmental researchers, which helps to broadly understand rock-water interactions and weathering processes based on association values of physio-chemical parameters, shown in Table 6. Furthermore, Fig. 2 represents the spatial distribution maps of WQI in pre (2015) and post monsoon (2016) period. The TDS values are plotted against the cation ratio (Na^+^ + K^+^) / (Na^+^ + K^+^ + Ca^2+^) and the anion ratio (Cl^-^) / (Cl^-^ + HCO_3_^-^), which is shown in Fig. 3. To infer the hydrochemical facies of groundwater, the piper [1] trilinear plot is presented in Fig. 4.The equations for calculating irrigation water quality indexes and ratios such as SAR, RSC, SSP, MHR, KR, %Na, PI, and PS are summarized in Table 7 and the outcomes are shown in Tables 8-9. The graph between Electrical Conductivity (EC) and percent sodium (% Na), while between EC and SAR is shown in Figs. 5 and 6, respectively. Similarly Fig. 7 classifies irrigation water in three classes.

**Table 1:** Charge balance error values.

Sample ID	%CBE	
	Post monsoon	Pre monsoon
LKO1	1.05	0.26
LKO2	1.42	-0.27
LKO3	2.03	0.02
LKO4	4.33	0.35
LKO5	-1.44	0.50
LKO6	-0.10	0.61
LKO7	4.86	-0.33
LKO8	-1.31	-4.26

**Table 2:** Laboratory and field observation of hydrochemical data of groundwater.

Sample ID	GPS Co-ordinate		pH	EC	TDS	Ca^2+^	Mg^2+^	Na^+^	K^+^	Cl^-^	SO_4_^2-^	HCO_3_^-^	TH	F^-^	NO_3_^-^
	**x**	**y**													
**2016 (Post-Monsoon)**															
LKO1	81.03	26.62	8	560	364	36	33	36	4.5	14	3	342	225.2	0.2	0
LKO2	81.12	26.74	8.1	462	300	40	28	16	2.8	7.1	16	268	215.2	0.6	0.34
LKO3	80.85	26.72	8.1	585	380	32	39	35	4.4	28	10	317	240.2	0.2	0.09
LKO4	80.94	27.04	8.5	520	338	20	30	49	5.6	18	12	275	175.1	0.7	0
LKO5	80.73	27.03	8.1	754	490	68	36	37	5.2	28	31	421	320.3	0.6	0.25
LKO6	80.69	26.93	8	615	400	48	41	20	3.7	14	17	366	290.2	0.7	1.2
LKO7	80.78	26.87	8.2	662	430	48	39	37	4.4	43	17	310	280.2	0.9	31
LKO8	80.94	26.86	8.2	738	480	60	36	37	4.8	57	46	325	300.2	0.3	108
**2015 (Pre-Monsoon)**															
LKO1	81.03	26.62	8.2	320	214	28	19	10	2.4	7	2.2	195	150	0.3	0.6
LKO2	81.12	26.74	8	614	411	36	34	48	2.8	21	19	354	230	0.1	1.9
LKO3	80.85	26.72	7.9	426	285	36	27	13	3.8	7	4.3	268	200	0	0.4
LKO4	80.94	27.04	8	591	396	32	32	51	4.8	21	6.3	354	210	0.2	0.3
LKO5	80.73	27.03	8.1	472	316	20	41	17	4	14	18	268	220	0.5	0.2
LKO6	80.69	26.93	7.9	567	380	36	36	28	5.8	7	2.5	354	240	0.2	0.3
LKO7	80.78	26.87	8.2	790	529	56	58	24	3.8	28	47	427	380	0.0	1.1
LKO8	80.94	26.86	7.9	710	473	61	37	35	4.7	59	45	355	310	0.3	106

Units of all the parameters expressed in mgL^-1^, except pH and electrical conductivity expressed in µmhos/cm.

**Table 3:** Assigned and relative weight for computing WQI as per BIS standards 2012.

Parameter	BIS standards (2012) (Desirable limit) mgL^-1^	Assigned Weight(wi)	Relative weight(RWi)
Calcium (Ca^2+^)	75	2	0.06
Magnesium (Mg^2+^)	30	2	0.06
Sodium (Na^+^)	200*	3	0.09
Potassium (K^+^)	12*	2	0.06
Nitrate (NO_3_^-^)	45	5	0.15
Sulphate (SO_4_^2-^)	200	3	0.09
Bicarbonate (HCO_3_^-^)	200	1	0.03
Chloride (Cl^-^)	250	3	0.09
Total Hardness (TH)	200	3	0.09
Fluoride (F^-^)	1	5	0.15
Total Dissolved Solid (TDS)	500	5	0.15
		Ʃ wi = 34	Ʃ RWi = 1

⁎ values are taken from WHO [5] guideline.

**Table 4:** Groundwater quality category based on WQI [6].

S No.	Range	Category	No. of Samples		Sample (%)	
			2015 (PreM)	2016 (PosM)	2015 (PreM)	2016 (PosM)
1	<25	Excellent water	0	0	0	0
2	25-50	Good water	6	4	75	50
3	50-75	Fair water	1	3	12.5	37.5
4	75-100	Poor water	1	1	12.5	12.5
5	100-150	Very poor water	0	0	0	0
6	>150	Unsuitable for drinking	0	0	0	0

**Table 5:** Groundwater quality index classification for individual sample based on WQI.

Sample No.	WQI	Water quality category
**2016 (Post-Monsoon)**		
LKO-1	41.9	Good water
LKO-2	42.0	Good water
LKO-3	44.7	Good water
LKO-4	45.3	Good water
LKO-5	60.5	Fair water
LKO-6	55.6	Fair water
LKO-7	68.9	Fair water
LKO-8	89.9	Poor water
**2015 (Pre- Monsoon)**		
LKO-1	27.4	Good water
LKO-2	43.5	Good water
LKO-3	32.1	Good water
LKO-4	43.4	Good water
LKO-5	43.2	Good water
LKO-6	44.8	Good water
LKO-7	58.6	Fair water
LKO-8	90.3	Poor water

**Table 6:** Correlation matrix between physico-chemical parameters of groundwater samples.

**Table 7:** Summary of irrigation water quality indices equations [10], [11], [12], [13], [14], [15].

S.No.	Indices	Acronym	Formula
1	Sodium Absorption Ratio	SAR	$\text{SAR}=\frac{\text{Na}}{\sqrt{\frac{\text{Ca}+\text{Mg}}{2}}}\;$
2	Residual Sodium Carbonate	RSC	$\text{RSC}=[\left(CO_{3}+HCO_{3}\right)-\left[\left(\text{Ca}+\text{Mg}\right)\right]$
3	Soluble Sodium Percentage	SSP	$\text{SSP}=\;[\frac{\text{Na}}{\left(\text{Ca}+\text{Na}+\text{Mg}\right)}]X\;100$
4	Magnesium Hazard Ratio	MHR	$\text{MH}=[\frac{\text{Mg}}{\text{Ca}+\text{Mg}}]X\;100$
5	Kelly's Ratio	KR	$\text{KR}=\frac{\text{Na}}{\text{Ca}+\text{Mg}}$
6	Percent Sodium	%Na	$\%\text{Na}=\frac{\text{Na}+K}{\text{Ca}+\text{Mg}+\text{Na}+K}\;X\;100$
7	Permeability Index	PI	$\text{PI}=\frac{\text{Na}+K+\sqrt{\text{HC}O_{3}}}{\text{Ca}+\text{Mg}+\text{Na}+K}\;X\;100$
8	Potential Salinity	PS	$\text{PS}=[\text{Cl}+(0.5\;x\;SO_{4})]$

**Table 8:** Calculated values of irrigation water quality indices.

Sample No.	SAR	RSC	SSP	MHR	KR	%Na	PI	EC	PS
**2016 (Post-Monsoon)**									
LKO-1	1.042544	1.09310	25.76386	60.18391	0.347053	27.14353	39.90986	560	0.427195
LKO-2	0.474628	0.09213	13.92992	53.58062	0.161844	15.14622	42.12295	462	0.366852
LKO-3	0.982082	0.38926	24.05626	66.77378	0.316764	25.38314	37.02259	585	0.889787
LKO-4	1.618882	1.04033	38.07333	71.21011	0.614813	39.61824	39.25198	520	0.632685
LKO-5	0.902807	0.54409	20.20551	46.60913	0.253219	21.51601	34.17892	754	1.112573
LKO-6	0.512214	0.22931	13.10341	58.48017	0.150793	14.32463	37.3364	615	0.571903
LKO-7	0.961408	-0.52391	22.30952	57.26096	0.287159	23.50288	32.48741	662	1.389956
LKO-8	0.932570	-0.63004	21.27167	49.73300	0.270191	22.52876	31.74922	738	2.086786
**2015 (Pre- Monsoon)**									
LKO-1	0.357500	0.23514	12.80944	52.80655	0.146913	14.3576	52.20749	320	0.220365
LKO-2	1.377554	1.20748	31.24545	60.89706	0.454449	31.9745	37.82385	614	0.790185
LKO-3	0.398933	0.37403	12.33633	55.29169	0.140723	14.15652	45.43549	426	0.242227
LKO-4	1.525357	1.57167	34.40141	62.24935	0.524423	35.62697	38.99621	591	0.65797
LKO-5	0.500141	0.02045	14.46701	77.17088	0.16914	16.14543	41.04004	472	0.582313
LKO-6	0.789556	1.04291	20.37757	62.24935	0.255928	22.30596	40.69097	567	0.223488
LKO-7	0.536684	-0.56907	12.12301	63.07037	0.137954	13.10378	31.5189	790	1.279143
LKO-8	0.872532	-0.27056	20.00236	50.00474	0.250037	21.24621	32.84148	710	2.132793

**Table 9:** Groundwater classification for irrigation use based on different irrigation indices.

Parameter	Range	Category	No. of Samples		Sample (%)	
			2015 (PreM)	2016 (PosM)	2015 (PreM)	2016 (PosM)
SAR	0-10	Excellent	8	8	100	100
	10-18	Good	0	0	0	0
	18-26	Doubtful	0	0	0	0
	>26	Unsuitable	0	0	0	0
RSC	<1.25	Good	8	8	100	100
	1.25-2.5	Doubtful	0	0	0	0
	>2.5	Unsuitable	0	0	0	0
SSP	<20	Excellent	4	2	50	25
	20-40	Good	4	6	50	75
	40-80	Marginal	0	0	0	0
	>80	Unsuitable	0	0	0	0
MHR	<50	Suitable	0	2	0	25
	>50	Unsuitable	8	6	100	75
KR	<1	Suitable	8	8	100	100
	1-2	Marginal	0	0	0	0
	>2	Unsuitable	0	0	0	0
%Na	<20	Excellent	4	2	50	25
	20-40	Good	4	6	50	75
	40-60	Permissible	0	0	0	0
	60-80	Doubtful	0	0	0	0
	>80	Unsuitable	0	0	0	0
PI	<80	Good	8	8	100	100
	80-100	Moderate	0	0	0	0
	100-120	Poor	0	0	0	0
EC	<250	Excellent	0	0	0	0
	250-750	Good	7	7	87.5	87.5
	750-2250	Permissible	1	1	12.5	12.5
	>2250	Doubtful	0	0	0	0
PS	<3	Suitable	8	8	100	100
	>3	Unsuitable	0	0	0	0

## 2 Experimental design, materials and methods

### 2.1 Study area description

Lucknow district is a flat alluvial area spread over about 2528 km^2^, located in the state of Uttar Pradesh, India, between latitudes 26°30′ to 27°10′ N and 80°30’ to 81°13’ E longitudes, and its elevation is about 103 m to 130 m amsl (Fig. 1). The sampling site has been chosen to collect the sample in such a way that it can give proper information about the ground water quality of the entire district. The Gomti River is mainstream of Lucknow district, flows from the central part of the district which splits the investigation area into two parts namely, Cis and Trans Gomti. Furthermore, Gomti-Ganga alluvial is divided into older and younger alluvium of quaternary age is the major geographic unit of the district. Older alluvium in the highland area composed of 3.3 to 6.5 ft. thick fine silty sand with scattered coverings of calcrete nodules while newer alluvium in lowland regions comprises silt, sand, and clay. Although, Central Ground Water Board (CGWB) dug several exploratory boreholes between 328 and 2470 ft. below ground level (bgl) and revealed that five aquifer groups exist in the area. In the present investigation area, both confined and unconfined aquifer systems are extensively used for domestic and irrigation use. In addition, the pre and post-monsoon depths of the water level are 17.06 to 127.28 ft. and 5.28 to 93.17 ft., respectively [2].

### 2.2 Sampling and Laboratorty analysis

Groundwater samples were collected from 08 shallow boreholes (Fig. 1). A total of 16 samples were collected (8 samples in pre monsoon and 8 samples in post monsoon) according to the standard procedure in 1L clean polyethylene bottles and noted the GPS coordinates of sampling point (Table 2) during the pre-monsoon (2015) and post-monsoon period (2016). The pH and EC were measured on site using PC/301, while Total dissolved solids (TDS) were measured using CB18/945 Generic hand-held TDS/3 digital meter. Total Hardness (TH) was determined by Ethylene Diamene Tetra Acetic Acid (EDTA) titrimetric method using Black-T indicator. Samples were filtered using cellulose filters (0.45µm) for determining the cations and anions using Ion chromatography (Metrohmn 792B-IC), which showed an accuracy of ±2%. Cations were measured using Metrosep C2/100 column such as Na^+^, K^+^ , Ca^2+^ , Mg^2+^ , while Metrosep A Supp 4/250 was used to measure the anions such as F^-^ , Cl^-^ , SO_4_^2-^ , NO_3_^-^ , HCO_3_^-^ . The charge-balance error was calculated to check the veracity of the chemical analysis using Eq. 1 and found to be within the allowable range of (±) 5% [3] which is presented in Table 1.

(1) $$\%\;\text{CBE}=\;\frac{\sum \text{TA}-\sum \text{TC}}{\sum \text{TA}+\sum \text{TC}}\;X\;100$$

## 3 Evaluation of groundwater quality index for drinking

The cumulative effect of different hydrochemical parameters on groundwater quality varies. The relative weight (RWi) of individual parameters has been calculated using Eq. (2):

(2) $$\text{RWi}\;\left(\text{relative}\;\text{weght}\right)=\;\frac{\text{wi}}{\sum_{i=1}^{n}\text{wi}}$$

Where, wi represents the assigned weight and n represents the number of parameters used in the analysis. The relative rate (RRi) of each parameter is computed using Eq. (3):

(3) $$\text{Relative}\;\text{rate}\;\left(\text{RRi}\right)=\;\frac{\text{ri}}{\text{BISi}}\;X\;100$$

Where, ri is ionic concentration of individual parameter, and BISi is the desirable limit recommended by BIS [4]

The WQI for each site is calculated by adding the standard index (SIi) values of the individual parameters using Eqs. (4) and (5), respectively:

(4) StandardIndex(SIi)=RWixRRi

(5) WaterQualityIndex(WQI)=∑SIi

### 3.1 Evaluation of groundwater quality indices and ratios for irrigation

In order to assess the quality of groundwater in relation to irrigation purpose, it is necessary to evaluate the composition and concentration of dissolved components [7], [8], [9]. Groundwater quality for irrigation purpose is explained on the basis of SAR, RSC, SSP, MHR, KR, % Na, PI, PS, and EC values are summarized in Tables 7-9. Wilcox [10], USSL [11], and Doneen [12] classifications are used to explain the suitability of groundwater for irrigation purposes shown in Fig. 5, Figs. 6 and 7, respectively.

## Declaration of Competing Interest

The authors declare that they have no known competing financial interests or personal relationships that could have appeared to influence the work reported in this paper.

## Appendix Supplementary materials

Image, application 1
